# Mental Health in Familial Adenomatous Polyposis: A Systematic Review

**DOI:** 10.1002/pon.70176

**Published:** 2025-05-12

**Authors:** M. J. Mol, H. Bouchiba, A. S. Aelvoet, F. Bennebroek Evertsz, F. A. M. Duijkers, J. G. Karstensen, B. A. J. Bastiaansen, E. Dekker, E. M. A. Bleiker

**Affiliations:** ^1^ Department of Gastroenterology and Hepatology Amsterdam UMC University of Amsterdam Amsterdam the Netherlands; ^2^ Cancer Center Amsterdam Amsterdam the Netherlands; ^3^ Amsterdam Gastroenterology Endocrinology Metabolism Amsterdam the Netherlands; ^4^ Division of Psychosocial Research and Epidemiology The Netherlands Cancer Institute Amsterdam the Netherlands; ^5^ Department of Medical Psychology Amsterdam University Medical Centres Amsterdam the Netherlands; ^6^ Department of Clinical Genetics Amsterdam Medical Center Amsterdam the Netherlands; ^7^ Danish Polyposis Registry Gastrounit Copenhagen University Hospital Amager and Hvidovre Hvidovre Denmark; ^8^ Department of Clinical Medicine University of Copenhagen Copenhagen Denmark; ^9^ Department of Clinical Genetics the Netherlands Cancer Institute Amsterdam the Netherlands; ^10^ Department of Clinical Genetics Leiden University Medical Center Leiden the Netherlands

**Keywords:** *APC* gene mutation, cancer, co‐occurrent psychiatric disorders, cognitive functioning, colorectal, disorders, familial adenomatous polyposis (FAP), mental health issues, oncology, SNP's

## Abstract

**Objective:**

Familial adenomatous polyposis (FAP) is a hereditary disposition causing a nearly 100% risk of developing colorectal cancer without treatment. Therefore lifelong endoscopic surveillance and prophylactic colectomy, generally before the age of 30, are offered. Children of genetically confirmed FAP‐patients have a 50% risk of inheriting the disease. It is a challenge to cope with the burden of FAP. The aim of this systematic review was to study the literature on the presence of mental issues, including anxiety, depression, distress, intellectual disability, and symptoms in the autism spectrum in FAP‐patients.

**Methods:**

A search was performed in PubMed, EMBASE and Cochrane Library to retrieve English studies on mental health and FAP from inception to May 2024.

**Results:**

Of the 2045 identified papers, 35 met our criteria. Most papers did not show increased levels in mean scores of distress, anxiety or depression. However, subgroups with high levels of distress, anxiety and depression were identified, specifically around the time of genetic testing and bowel surgery. Associations between FAP and co‐occurrent psychiatric disorders and lower cognitive function were suggested.

**Conclusions:**

In conclusion, there is some evidence that co‐occurrent psychiatric disorders and intellectual deficits are more prevalent in FAP‐patients compared to the general population. During genetic testing and bowel surgery, distress, anxiety and depressive symptoms in subgroups of FAP‐patients might be increased. As FAP is a rare disease and sample sizes were small, firm conclusions cannot be drawn. Large prospective studies on mental health issues over time are needed.

## 1 Background

Familial adenomatous polyposis (FAP) is an autosomal dominant inherited condition, characterized by the development of hundreds to thousands of polyps throughout the colon and rectum. In the majority of FAP patients, a pathogenic variant in the adenomatous polyposis coli (*APC*) gene can be detected. However, in a subset of patients who present with more than 100 colorectal polyps, no pathogenic variant in the *APC* gene can be identified. These patients have a clinical diagnosis of FAP instead of a confirmed genetic diagnosis.

When left untreated, nearly 100% of all FAP‐patients develop colorectal cancer (CRC), which typically occurs between the age of 35–45 years [1]. Once the diagnosis is confirmed by genetic testing, patients may undergo prophylactic colorectal surgery to prevent CRC. The preferred surgical procedures for FAP are a restorative proctocolectomy with ileal pouch‐anal anastomosis (IPAA) or a subtotal colectomy with ileorectal anastomosis (IRA) or ileosigmoidal anastomosis (ISA). The timing of prophylactic surgery differs from the late teens to the early thirties. However, indications and timing of prophylactic surgery in FAP‐patients differ between clinics.

Individuals with a family history of FAP are recommended to undergo genetic counseling and screening for FAP between the young age of 10 and 12 to identify if they have a pathogenic variant in the *APC* gene [2, 3]. Although it is recognized that having the genetic predisposition to develop FAP can impact quality of life (QoL) [4], there are no guidelines on how to address psychosocial issues or how to provide mental support. In combination with the physical burden of undergoing (multiple) surgery (‐ies), regular surveillance and being at risk of developing cancer, this might lead to FAP‐patients developing mental health issues. Furthermore, a family burden, such as losing family members due to cancer, can lead to a wide variety of issues [5].

The impact of being diagnosed with FAP might result in mental issues. Some studies reported that approximately 15%–20% of FAP‐patients had moderate to severe levels of FAP‐specific distress [4, 6]. Significantly more individuals with a diagnosis of FAP had frequent cancer worries than those being tested for FAP or those that tested negative (non‐carriers). Psychosocial issues including stress, symptoms of anxiety, depression, fear (perceived cancer risk) and negative body image are present in up to 72% of FAP‐patients [7]. One study described that 11% of all FAP‐patients met all four of the American Psychiatric Association diagnostic criteria for posttraumatic stress disorder (PTSD), and 10.1% endorsed partial PTSD criteria with 3 of 4 symptoms [7]. These factors seem to be in accordance to the DSM V adjustment disorder subtypes, ‐with depressed mood, ‐ with anxiety and ‐with anxiety and depression. Adjustment disorder are characterized by excessive reactions to stress that involve negative thoughts, strong emotions and changes in behavior [8]. In this case, being confronted with (living with) FAP might induce such a stress reaction.

Since the patient journey of the typical FAP‐patient is challenging, it is difficult to distinguish the exact causes of mental health issues. Are mental health issues being caused by the FAP related stressors or does the pathogenic variant in the *APC* gene form a predisposition for developing mental issues, or is it a combination of the two? Zhou et al. [9] showed that common variants (SNPs) in the *APC* gene might be associated with Autism Spectrum Disorder (ASD) and Cui et al. [10] showed that genetic variation in the *APC* gene was associated with susceptibility to schizophrenia. These results suggest that a pathogenic variant of the *APC* gene might also be related to co‐occurrent psychiatric disorders.

Additionally, in a systematic review Privitera et al. [11] evaluated the association between having FAP and Intellectual disability (ID). The review considered FAP‐patients with a chromosome abnormality (a deletion of multiple genes including the *APC* gene) and concluded that the KCNN2 gene (5q22.3 located near the *APC* gene) is the most likely candidate gene contributing to ID in FAP‐patients.

In this systematic review, we aim to evaluate which specific mental health issues FAP‐patients may encounter during their life. More specifically, we aim to investigate mental issues associated with the psychological adjustment of the individuals to their new diagnosis, as well as their co‐occurrent psychiatric disorder. Furthermore, IQ and ID in FAP‐patients will be evaluated.

## 2 Methods

This systematic review is performed in accordance with the Preferred Reporting Items for Systematic Reviews and Meta‐analyses (PRISMA) guidelines. The review protocol was published in the PROSPERO international register of systematic reviews (CRD42023430971).

### 2.1 Study Outcomes and Definitions

We assessed the following outcomes in patients with FAP: adjustment issues, including distress and symptoms of anxiety and/or depression; co‐occurrent psychiatric disorder, such as anxiety disorders, depressive disorders, PTSD, schizophrenia, bipolar disorder and ASD; and (neuro) cognitive alterations, development delay, IQ and ID.

The term “distress” is broadly used in literature to define the mental state of individuals. Since comparisons between scores resulting from a wide variety of scales is challenging, we divided distress into two main components: stress and cancer worries. Other definitions used by authors of included papers are also investigated and evaluated on their usability in this review.

The terms “anxiety symptoms” and “depressive symptoms” are defined by the score on screening instruments that assess a global anxiety or depression score. Official diagnoses such as Generalized Anxiety Disorder (GAD) or Major Depressive Disorder (MDD) will be considered in the co‐occurrent psychiatric disorder section.

In this systematic review, additionally to IQ, ID is investigated. ID involves problems with general mental abilities that affect functioning in two different areas: 1) intellectual functioning (such as learning, problem solving) which can be assessed by an IQ score, and 2) adaptive functioning (daily life activities, such as independent living and communication).

### 2.2 Search Strategy

With the assistance of an information specialist, we performed a systematic search using Pubmed, EMBASE and the Cochrane Library to identify studies up to February 2023. We searched for terms including FAP and APC gene mutation to identify studies about FAP‐patients and their pathogenic variant, and terms to identify mental issues such as distress, anxiety, depression, post‐traumatic stress disorder, schizophrenia, bipolar disorder, cognitive functioning and mental retardation. No restrictions on publication date were applied. Only English publications were searched. Potential relevant articles were further identified by cross‐referencing. The search was updated on the 21th of May, 2024. More details on the search strategy can be found in Supporting Information S1: Appendix A.

### 2.3 Study Selection

Studies were considered eligible when they included outcomes on distress, symptoms of anxiety and/or depression, co‐occurrent psychiatric disorders or intellectual functioning. No age restrictions were set. Studies were excluded when no conclusions could be drawn for FAP‐patients specifically, for example if half the sample consisted of FAP‐patients and half of ulcerative colitis patients and conclusions were drawn for the whole study sample only.

Two reviewers (MM, HB) independently screened all titles and abstracts of the identified studies using the online systematic literature review software Rayyan [12]. Studies considered potentially eligible were included or excluded based on a corresponding full text screening. Conflicts in decisions between reviewers were resolved through discussion.

### 2.4 Data Extraction

The two reviewers independently extracted data from the included studies. They used a standardized data extraction form, extracting the following study characteristics: authors, year of publication, country, study design, sample size, study population (speaking language, families etc.), response rate, inclusion/exclusion criteria, type of mutation, de novo versus familial, age, gender, adaptive outcomes, co‐occurrent psychiatric disorders, cognition outcomes, support need outcomes, type of outcome, instrument, timepoint in the patient journey, time points of study, control group, group interactions and limitations. After data from the first five studies had been extracted, the two reviewers discussed the outcomes to create the final extraction form. After consensus was reached and adjustments were made to the extraction form, one reviewer (MM) extracted the remaining studies according to the final extraction form. Afterward, the second reviewer (HB) extracted five randomly selected papers and results were discussed between the two reviewers.

### 2.5 Risk of Bias Assessment

The same two reviewers critically appraised the first five studies. After discussion and agreement among the two reviewers, one reviewer (MM) critically appraised the remaining studies. Afterward, the second reviewer (HB) critically appraised five randomly selected papers and results were discussed. Three separate critical appraisal tools were used. (1) The Newcastle Ottawa Scale (NOS) for cohort and case‐control studies [13], (2) the Appraisal tool for Cross‐Sectional Studies (AXIS) [14] and (3) the checklist from Boejie et al. [15].

### 2.6 Statistical Analysis

In this systematic review, we evaluated the impact of FAP on levels of distress, symptoms of anxiety and/or depressive symptoms. We investigated the associations with co‐occurrent psychiatric disorders, cognitive differences, and ID. Since different instruments were used and outcome measures could differ per study, no statistical analyses of the results were performed. However, when enough information was reported, effect sizes of statistical analyses were calculated by the main author (MM).

## 3 Results

### 3.1 Study Characteristics

**FIGURE 1:**
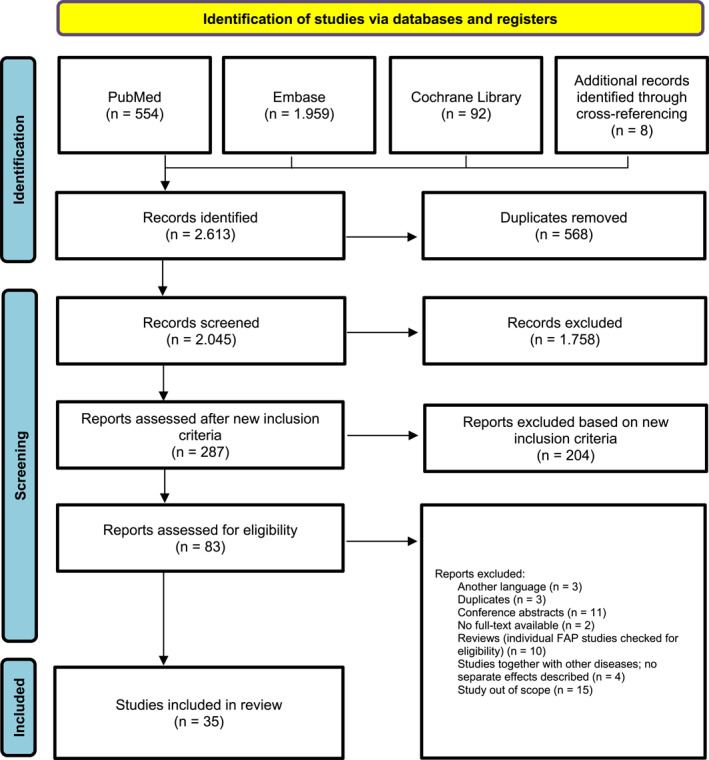
PRISMA‐flowchart.

First, 2.605 studies were identified with the search. Through cross‐referencing 8 articles were added leading to a total of 2.613 records. After removing duplicates, 2.045 were screened for eligibility based on title and abstract and 287 studies were identified. After careful consideration, we decided to remove QoL as a selection criterium as the majority of the identified studies focused on QoL after bowel surgery and the impact of FAP‐related surgeries on QoL is already known [16, 17]. We decided to focus on psychiatric issues in FAP‐patients. We have taken into account both symptoms of anxiety and/or depressive symptoms and disorders of anxiety and depression. In total 83 studies about mental health issues in FAP‐patients were identified and screened for full text, ending up with 35 studies (Figure 1). We found no randomized controlled trials, 14 manuscripts reporting cross‐sectional data, 2 cohort studies, ten case‐control studies and 13 qualitative studies. Study characteristics are summarized in Supporting Information S1: Table 1 in Appendix B. A total of 16 studies were conducted in Europe, 16 in North‐America, 4 in Australia and 3 in Asia. By what means studies investigated variables is reported in Supporting Information S1: Appendix B under column “Type of study” and in Supporting Information S1: Appendix C under column “Measure”. Individual disease characteristics such as polyp burden, surgery, cancer (history) and bowel function is reported in Supporting Information S1: Appendix C under column “Individual disease characteristics”.

### 3.2 Risk of Bias

The results of the risk‐of‐bias assessment are shown in Supporting Information S1: Table 1, 2, 3 and 4 in Appendix D. Selection bias might be present in some identified studies, since these studies recruited their participants via a registry, while we should acknowledge that not all patients might be filed in such registries. Four studies did not use validated questionnaires [4, 7, 18, 19]. The response rate was sometimes low or not given [7, 9, 10, 20, 21]. The inter‐rated reliability between the two reviewers was 91%. No major differences in extracting the results were identified, thus there is no reason for concern in the quality of data extraction.

### 3.3 Adjustment Issues: Distress and Symptoms of Anxiety and/or Depression

#### 3.3.1 Instruments

As shown in Supporting Information S1: Table 1 in Appendix C, in the identified studies about distress the Impact of Event Scale (IES) [22] and the Cancer Worry Scale (CWS) [23] were most commonly used. Other instruments were the Children's Global Assessment Scale (CGAS) [24] and self‐created questionnaires, making a comparison between the studies challenging.

As shown in Supporting Information S1: Table 2 in Appendix C, anxiety has been measured by the Spielberg State‐Trait‐Anxiety‐Index (SSTAI) [25], the Hospital Anxiety and Depression Scale (HADS) [26], the Revised Children's Manifest Anxiety Scale (RCMAS) [27] and self‐created questionnaires.

As shown in Supporting Information S1: Table 3 in Appendix C, the instruments evaluating depression were the Children's Depression Inventory (CDI) [28], the Beck Depression Index (BDI) [29], Reynold's Adolescent Depression Scale (RADS) [30] and the Hospital Anxiety and Depression Scale (HADS) [26].

#### 3.3.2 Timepoints

The included studies were inspected and a clear division was identified. The papers investigating distress, anxiety or depression mainly focused on (1) either the whole patient journey of a FAP‐patient or one of two specific timepoints: (2) the genetic testing or (3) the bowel surgery. We will use these three categories to discuss distress, anxiety and depression. A summary of the results for this section is shown in Table 1.

**TABLE 1:** Distress, anxiety and depression in FAP patients by moment in the patient journey.

	Moment	Significant differences^a^	Subgroups identified	Contradicting results	Non‐significant^a^	Total	Papers per topic
		*N*	*N*	*N*	*N*	*N*	*N*
Distress	Any timepoint	0	1	0	0	1	9
	Genetic test	1	2	0	3	6	
	Bowel surgery	1	1	0	0	2	
Anxiety	Any timepoint	0	0	0	0	0	8
	Genetic test	1	3	0	2	6	
	Bowel surgery	1	0	1	0	2	
Depression	Any timepoint	0	0	0	1	1	7
	Genetic test	0	1	0	4	5	
	Bowel surgery	0	0	1	0	1	
		4	8	2	10	24	

^a^ refers to the results of tests of disparity in mean levels of distress, anxiety or depression between groups (e.g. the mean anxiety level was found to be significantly higher for FAP patients as compared to the general population).

#### 3.3.3 Adjustment Issues: Distress, Anxiety and Depression not Related to any Timepoint in the Patient Journey

One study investigating distress in FAP families through any time point in their lives showed no significant increase in mean distress levels in FAP‐patients [6]. However, approximately 20% of the respondents had clinically relevant levels of FAP specific distress. There were significantly more individuals in the FAP group having frequent cancer worries than individuals in the at risk of FAP group and the non‐carrier group. This study provides quite strong evidence for the presence of higher distress levels in a subset of FAP‐patients, but shows no increased levels of distress for the entire FAP population. The study also took history of surgery and personal cancer history into account and both were not associated significantly with cancer worries in multivariate analyses.

We found no studies on anxiety in FAP‐patients investigating anxiety at a specific timepoint in the patient journey.

One study reported that FAP‐patients scored in the normal range of depression according to the DBI [31].

#### 3.3.4 Adjustment Issues: Distress, Anxiety and Depression in Relation to Genetic Testing

Of the studies investigating distress surrounding genetic tests, shown in Supporting Information S1: Table 1 in Appendix C, one study found a lower level of psychosocial functioning in children with FAP as measured by the CGAS, with a large effect size (Cohen's D: −1.55) [32].

Two studies showed no significant group differences, but rather identified a subgroup with increased distress levels. The study of Michie et al. [33] showed no increase in distress in children directly after genetic testing. However, 33 weeks after receiving their test results, those receiving a positive result were more distressed about FAP in the family than those receiving a negative result. Andrews, Mireskandari, Jessen, Thewes, Solomon, Macrae, and Meiser [4] found no significant difference in mean distress levels after the genetic test for FAP patients. However, 4.5% and 11.4% of the participants had stress scores on the subscales of the IES indicative of a significant stress response.

Three studies conclude non‐significant differences in mean levels of distress [33, 34, 35] of which one had a small effect size [33], and one was specifically about presymptomatic testing [34].

When investigating the studies on symptoms of anxiety surrounding the genetic test, Michie et al. [33] showed that positive test results were related to increased anxiety levels in both children (effect size D: 0.52, medium) and adults (effect size D: 0.83, large). In total 19% of children versus 43% for adults had anxiety scores in the clinical range.

Three studies showed no significant group differences, but rather identified a subgroup with increased anxiety levels [33, 36, 37]. Codori, Petersen et al. [36] found that all levels of anxiety in participating children were subclinical. However, regardless of the genetic test result, children with mothers with FAP had increased anxiety scores 3 months later at follow‐up while children with affected fathers had decreased scores. Codori, Zawacki et al. [37] found no clinically significant psychiatric levels of anxiety at any time point during the follow up of 2–4 years. Although most children had no clinically significant declines in their psychological functioning after genetic testing for FAP, the non‐FAP siblings of children who tested positive appear particularly vulnerable to clinical levels of anxiety symptoms after testing. The follow‐up study of Michie et al. [33] showed SSTAI anxiety scores of children tested positive for FAP were within the normal range of anxiety and children showed a decrease in anxiety after receiving a negative test result. At the second post‐test assessment, those receiving a positive result were more anxious than were those receiving a negative result. This study included 31 participants.

Two studies with 45 and 23 FAP patients showed no significant increase in anxiety levels [34, 38].

When investigating the studies about depression during the genetic tests, a study about depressive symptoms in children being tested for FAP and their parents found no significant group differences between children being tested positive and negative [37]. No differences in diagnosing depression for neither children nor parents were found. It rather identified a subgroup with increased depression levels. A positive test and having a positive sibling appeared associated with significant, but subclinical, increases in depression symptoms.

Four studies indicated no increased depression levels in FAP‐patients, children with FAP, parents and partners [33, 34, 36, 38]. One study [34] included 23 participants and another study [33] had a low effect size for adults and a medium effect size for children (Supporting Information S1: Appendix E).

#### 3.3.5 Adjustment Issues: Distress, Anxiety and Depression in Relation to Bowel Surgery

When investigating distress at time of bowel surgery, 1 study found that 40% of 89 participants having undergone an IRA had a high level of worries about their future health [18].

Durno et al. [39] found that one third of FAP‐patients having undergone bowel surgery at an early age, reported cancer worries after surgery, and again a small subset of patients experienced significant levels of distress. This study included 32 participants.

When investigating the studies about anxiety at time of bowel surgery, 1 study of 89 FAP patients having undergone an IRA found an increased level of anxiety [18]. It reported an initial anxiety response to the diagnosis of FAP in 52% of the patients. No information on the used questionnaire was provided and this study was performed in 1986.

In 2008, a small study in 21 patients having undergone an IPAA of Osterfeld et al. [40] reported contradicting results on anxiety and depression outcomes. They described 38% of patients reporting considerable fear and depressed mood shortly before the operation, and nearly all (90%) being afraid for an ileostomy. However, the level of anxiety as measured by the HADS remained unchanged over time; at no time period did patients' mean levels differ from those of the normal population after adjustment for sex and age.

In conclusion, there is no evidence for increased levels of distress and depression in the entire FAP population at any point in time, however, distress levels are higher in some subgroups. Additionally, there is some indication that distress, anxiety and depression levels are increased at the time of genetic testing in a subgroup of FAP‐patients. Finally, there is some evidence for increased distress and anxiety around the bowel surgery.

#### 3.3.6 Distress in Partners of FAP‐Patients

Additionally, the data on partners of FAP‐patients show conflicting results. Douma, Bleiker et al. [41] concluded no increased level of distress on group level, but 30% of the partners experiences clinically relevant levels of distress, whereas DudokdeWit, Tibben, Duivenvoorden, Niermeijer, and Passchier [34] indicates no significant increases in distress in partners.

### 3.4 Co‐Occurrent Psychiatric Disorders

The studies on co‐occurrent psychiatric disorders in FAP can be categorized either as empirical or genetic studies.

As shown in Supporting Information S1: Table 4 in Appendix C, four studies were considered of empirical nature. Wood et al. [7] indicated a high prevalence of PTSD. The incidence of PTSD was higher for FAP patients having undergone an IPAA. Gjone et al. [32] concluded that children aged 15 to 20 who inherited FAP from a parent more often had co‐occurrent psychiatric disorders (37%) than the general population (14%). This study included only 22 participants. In the psychiatric disorder group, significantly more chronic family issues were present than in the non‐psychiatric disorder group. Levitt et al. [31] found 34% of participants had either major depression, minor depression, GAD or schizophrenia. Gorrepati et al. [42] found that 12% (4/33) FAP‐patients had anxiety and/or depression, which is not significantly higher than the general population. In a recent study with a larger sample size in Denmark, a higher prevalence for psychiatric contacts, ‐prescriptions, ‐diagnoses and ‐events in FAP‐patients compared to the general population was found [43]. Additionally, FAP‐patients diagnosed due to symptoms or CRC without previous knowledge of a hereditary disease in the family and at‐risk patients having a family history of FAP were evaluated separately to the general population. FAP‐patients without previous knowledge of the disease did not have a significantly increased risk of psychiatric events, ‐diagnoses, ‐prescriptions, or ‐contacts compared to the general population. These risks were significantly increased for FAP‐patients with a known family history of FAP. When adjusting for age, sex and the event of cancer, the risk for a mood or behavioral and emotional disorder was significantly higher for FAP‐patients than the general population. No significant difference was found for schizophrenia, neurotic, or developmental disorders. While the risk of mood or neurotic disorders was significantly increased for FAP‐patients having a known family history of FAP, this was not the case for the patients without a known family history.

Although only evaluated in a few studies, there is some evidence that co‐occurrent psychiatric disorders are more prevalent in FAP‐patients compared to the general population. More research on this topic is needed.

We identified three studies evaluating a possible association between the *APC* gene and psychiatric disorders. Cui et al. [10], Yang et al. [21] and Zhou et al. [9] have investigated the influence of single nucleotide polymorphism (SNPs) and haplotypes on the incidence of schizophrenia, MDD and ASD respectively. All studies had sufficient sample sizes, although they used a candidate gene approach with often insufficient correction for population stratification. Cui et al. [10] demonstrated that three SNPs within the *APC* gene and three *APC* haplotypes were significantly associated with schizophrenia, suggesting that the *APC* gene may be a candidate gene conferring susceptibility to schizophrenia. Yang et al. [21] showed that two SNPs within the *APC* gene exhibited a statistically significant association with MDD. These results suggest that the *APC* gene may be one of the susceptibility genes for MDD and forms a genetic link between psychiatric disorders and cancer. Lastly, Zhou et al. [9] investigated if a previously identified SNP in the coding region within the *APC* gene was associated with ASD by genotype and allele frequency. They showed a significant association between the SNPs and ASD. Additionally, one haplotype at the *APC* locus was significantly associated with ASD.

These results suggest that common variance in the *APC* gene might contribute to schizophrenia, MDD and ASD in FAP‐patients (Supporting Information S1: Table 4 in Appendix C). Further research on the significance of biologic presence of the disease (pathogenic *APC* variant) is needed.

### 3.5 Cognitive Abilities

As shown in Supporting Information S1: Table 5 in Appendix C, four studies evaluated IQ and one study the highest level of education in FAP‐patients. Three studies indicated a lower cognitive function, where two did not. Of the latter, Azofra et al. [44] compared only three FAP adolescents and their non‐FAP age‐ and gender‐matched siblings. Levitt et al. [31] suggests the FAP group, consisting of 38 patients, scored an average level on the Shipley Institute of Living Scale (SILS) [45], and the Digit Symbols subtest of the WAIS. However, the de novo FAP group seemed to score somewhat higher on the intelligence scales than the inherited FAP group.

Results by Cali et al. [20] suggested a lower IQ in 18 FAP‐patients as compared to 16 healthy controls. Cruz‐Correa et al. [46] found significantly lower performance on IQ and in a variety of neurocognitive functions in FAP patients as compared to age, gender and educational level matched controls. Effect sizes were within the large range (Supporting Information S1: Appendix E). In a large cohort study in Denmark, the highest attained level of education was significantly lower for FAP‐patients than for the general population [43]. It is important to note that the disease might have influenced career and school choices. Though having a lower level of highest education attained can be an indication of a lowered cognitive ability, we cannot conclude a causal relationship.

Only one of the four papers on IQ scores reported a large effect size [46] and this paper found a significantly lower IQ score for FAP‐patients as compared to the age, gender and educational level matched controls. These papers provide the strongest indication of FAP‐patients having a lower cognitive function. However, none of these studies excluded patients with a larger deletion than only the *APC* gene, and maybe those patients were also included in the cohorts. This might have influenced the results significantly.

### 3.6 Intellectual Disability

Eleven case reports about ID in FAP‐patients were identified [47, 48, 49, 50, 51, 52, 53, 54, 55, 56, 57]. These were all patients with a large deletion entailing multiple genes including the *APC* gene. The neurodevelopmental/ID phenotype in these patients is most likely caused by other gene(s) than the *APC* gene. To our knowledge, apart from the systematic review [11] discussed in the introduction section, no studies investigating the prevalence of ID in FAP‐patients have been published and this paper only involved patients with a larger deletion. No studies were identified in patients without a larger deletion involving other genes so we cannot conclude whether ID is more common in FAP‐patients.

## 4 Discussion

In this systematic review, we identified three fluctuating mental health outcomes depending on the moment in the patient journey: adjustment problems concerning distress and symptoms of anxiety and/or depression. When comparing the group of FAP‐patients with the general population, we saw no significant differences in levels of distress, anxiety, or depression. However, in a subset of patients and at specific moments in life, namely at the time of genetic testing and the bowel surgery, increased levels of distress and anxiety were detected. Additionally, current literature suggests co‐occurrent psychiatric disorders are more prevalent in FAP‐patients, possibly associated with the SNP variants in the *APC* gene. While FAP‐patients may have a lower cognitive score, ID cannot be reliably assessed as we could identify only a few case reports on this topic.

None of the included studies examined differences in mental health issues between clinically and genetically diagnosed FAP patients. Comparing the mental health status between FAP patients with and without the identification of the exact disease causing pathogenic variant could provide valuable insights into the underlying etiology of mental health issues.

Only two studies reported on both adjustment issues and co‐occurrent psychiatric disorders and neither investigated a possible association between them [31, 32]. It would be interesting to investigate whether psychiatric disorders are related to higher levels of adjustment issues in FAP‐patients.

We evaluated adjustment problems in the form of non‐diagnosed levels of distress, anxiety and depression in FAP‐patients. The included studies on these topics often used different screening instruments and the definition of “distress” varied between studies (e.g. psychological distress, distress, FAP‐specific distress, cancer worries). Thus, different conclusions were drawn under the umbrella term ”distress”. We clearly advocate for consistent use of clear definitions and well‐framed terminology when investigating measures of mental health.

It is important to note that distress is common, ranging from normal distress for having FAP and undergoing medical surveillance and treatment to the diagnosis of anxiety disorders (including PTSD) and depressive disorders. It is therefore hard to distinguish what levels of distress are problematic and will lead to more extensive issues.

Most studies evaluating symptoms of anxiety and/or depression in FAP‐patients by self‐report, for example using the HADS. It should be noted that the HADS is not a diagnostic instrument but rather screens for anxiety and depressive symptoms. A more appropriate method to assess mental health disorders would employ clinical interviews to diagnose mental health disorders, such as the structural clinical interview for DSMV Axis 1 Disorders (SCID‐I) [58] or the World Health Organization (WHO) Composite International Diagnostic Interview (CIDI) [59]. Another method would be to assess mental health disorders based on chart reviews in medical files.

Karstensen et al. [43] found a significantly increased risk of psychiatric events, psychiatric diagnoses, needs for prescription of psychiatric medication, or psychiatric contacts for FAP‐patients with a family history of FAP as compared to the general population, while FAP patients being diagnosed due to symptoms or CRC without previous knowledge of and experience with the disease did not. This might suggest that the pathogenic variant in the *APC* gene does not cause a predisposition to developing psychiatric disorders and this increased risk is due to having to live with the disease and its interference with day‐to‐day life. Moreover, these issues might arise also on family level due to the burden of having a chronic disease in the family. Individuals born in FAP families are faced with the disease very early on in their lives. In these families, issues surrounding FAP do not only start at the moment individuals are diagnosed with the disease (usually tested at around 10–12 years of age), but these families carry a burden throughout their lives. As previously highlighted in this review, non‐FAP siblings of FAP children appear particularly vulnerable to clinical levels of anxiety symptoms after testing [37]. A subset of partners of FAP patients, who are integral to the family system, also exhibit clinically significant levels of distress [41]. Additionally, in Gjone et al. [32], FAP patients with psychiatric disorders had significantly more chronical family issues than FAP patients without psychiatric disorders. In the future, large international studies are needed which use a prospective longitudinal study design, to investigate the impact on family systems in larger cohorts.

The results in this review on cognitive abilities indicate a significantly lower IQ and level of education in FAP‐patients. This might be due to having had less opportunities for individuals with FAP. Historically, FAP families had a higher prevalence of early deaths, distorting the family dynamics and introducing traumatic experiences. Additionally, FAP patients undergo extensive surveillance and prophylactic surgery, potentially interfering with a focus on achieving educational goals.

When evaluating ID, we found many case reports. The prevalence of ID in FAP‐patients was not assessed in quantitative studies, potentially explained by a low prevalence of ID in FAP or a too small impact on cognition to quantitatively investigate if the *APC* gene is indeed responsible for causing ID.

Most studies included in this systematic review do not report or consider individual disease characteristics, such as polyp burden, history of surgery, cancer history, and bowel function, as potential factors influencing the mental health of FAP patients. To understand the mental state of FAP patients, their complex individual situation, including the intensity of the disease and the burden of having a chronic disease in the family should be taken into account.

### 4.1 Limitations and Strengths

A limitation of this study is that many of the included studies had small sample sizes and a low participation rates, which might have led to a non‐response bias. Another limitation is the small number of studies per topic, making it difficult to draw firm conclusions. Furthermore, selection bias may have played a role, as patient populations were often taken from patient registries that might not have registered all FAP‐patients in a population. Included studies mostly investigated FAP‐patients with a wide age‐range. The patient journey is very different for each individual FAP‐patient, as they all may have different major and minor disease events. As the life of an FAP‐patient is full of factors that may influence mental health, it is nearly impossible to control for all these events. Examples are type of colectomy, age of colectomy, timing of colectomy relative to other major life events (e.g. final Exam week, advancing from primary school to high school, etc.), repeat surgical and non‐surgical interventions for other (extracolonic) FAP related manifestations, timing of and age at genetic testing, family issues, risk of cancer, fecal continence, FAP‐related relational issues, social issues and financial issues. This might result in a large variability in mental health outcomes. In an effort to control for these impactful and variable factors, longitudinal research is needed. Since the variability is high among FAP‐patients, generalizing their results to the wider population should be done with caution.

In this review, only few studies found medium to large effect sizes and therefore demonstrated clinical significance. While most studies contributed to the broader understanding of the topic, the findings were often not robust enough to suggest practical applications in clinical practice. More research with larger sample sizes and power, possibly achieved through international collaboration, is needed.

A strength of this systematic review is the performed quality assessment, capturing aspects of the reviewed papers that could potentially alter their impact on the conclusion. In addition, this review covers a combination of mental issues, such as distress, anxiety and depression together with co‐occurrent psychiatric disorders, which are related and influence each other. Besides, a combination of qualitative and quantitative research has been included in this review.

### 4.2 Clinical Implications and Future Recommendations

It is clear that more research is needed. We propose an extensive questionnaire study in a large FAP population including siblings and partners, taking into account individual disease characteristics. Since FAP is a rare disease, this might have to be performed in an international collaboration. Investigating large FAP samples will enable adequate evaluation of, for example, the possible genetic associations between FAP and co‐occurrent psychiatric diseases or intellectual deficits. Regular screening for mental health issues can then be performed to ensure early diagnosis and optimal treatment. For the FAP‐patient this will mean filling in additional mental health questionnaires and having the possibility for an intake by a medical psychologist specialized in FAP. Only a small subset of FAP‐patients frequently makes use of healthcare services and therefore exhaust a large portion of the healthcare resources. When mental issues are preemptively dealt with, healthcare resources can be distributed more evenly. This may be done through extra care from medical staff or by implementing collaborative care. This includes embedding the availability of a social worker, medical psychologist specialized in FAP‐care or professional patient navigator in the patient journey, as in done for other diseases such as breast cancer and melanoma patients [60].

## 5 Conclusion

In conclusion, the results of our systematic review point to different directions. Although studies are scarce, there is some evidence that co‐occurrent psychiatric disorders and intellectual deficits are more prevalent in FAP‐patients compared to the general population. To draw firm conclusions, more research in larger samples of FAP‐patients is needed. Patients with large gene deletions involving more genes than the *APC* gene alone should be excluded from those studies. For distress, anxiety and depression, research should take into account the moment in the patient journey, specifically during the genetic testing (including siblings) and bowel surgery, and besides, it would also be interesting to study the partners of FAP‐patients. During these moments, distress, anxiety and depressive symptoms might be increased, and therefore offering timely assistance might be crucial for patients to address these issues and prevent future problems.

## Author Contributions

A.A., B.B., E.D. and E.B. designed the concept of the study. M.M. and H.B. screened the included studies, performed the data extraction and risk of bias assessment. M.M. wrote the manuscript. All authors critically revised the manuscript. All authors have approved the final manuscript.

## Conflicts of Interest

Mathijs J. Mol: none to declare. Hicham Bouchiba: none to declare. Arthur S. Aelvoet: none to declare. Floor Bennebroek Evertsz: none to declare. Floor A.M. Duijkers: none to declare. John G. Karstensen: consultancy for Boston Sci, SNIPR BIOME, and AMBU. Speakers' fee from Norgine. Barbara A.J. Bastiaansen: speaker’s fee from Olympus, Tillots Pharma A.G., Ovesco Endoscopy A.G. Evelien Dekker: Endoscopic equipment on loan of FujiFilm, and received a research grant from FujiFilm. Honoraria for consultancy from Olympus, Fujifilm, Ambu, InterVenn, Norgine, and Exact Sciences. Speakers' fees from Olympus, GI Supply, Norgine, IPSEN/Mayoly and FujiFilm. Eveline M.A. Bleiker: none to declare.

## Code Availability

The code in which effect sizes are calculated for this study are available from the corresponding author (E.B.) upon request.

## Supporting information

Suporting Information S1

## Data Availability

The data that support the findings of this study are available from the corresponding author (E.B.) upon request.
