# Supplementary material for: Mental Health in Familial Adenomatous Polyposis: A Systematic Review
Source: Psychooncology. 2025 May 12;34(5):e70176. doi: 10.1002/pon.70176 (PMC12069740; doi:10.1002/pon.70176)
Supplement: Supplementary file 1 — Suporting Information S1 [file PON-34-e70176-s001.docx]

Appendix A: Search strategy

PUBMED

| # | Searches PUBMED |
| --- | --- |
| #1 | ("Adenomatous Polyposis Coli"[Mesh] OR "Genes, APC"[Mesh] OR "Adenomatous Polyposis Coli Protein"[Mesh] OR adenomatous polyposis col*[tiab] OR APC gene*[tiab] OR protein FAP[tiab] OR familial adenomatous polypos*[tiab] OR familial polyposis col*[tiab] OR adenomatous intestinal polypos*[tiab] OR familial intestinal polypos*[tiab]) |
| #2 | ("Mental Disorders"[Mesh] OR "Depression"[Mesh] OR "Psychological Distress"[Mesh] OR "Behavioral Symptoms"[Mesh] OR "Psychological Well-Being"[Mesh] OR "Anxiety"[Mesh] OR "Sadness"[Mesh] OR "Stress, Psychological"[Mesh] OR "Shame"[Mesh] OR "Stress Disorders, Post-Traumatic"[Mesh] OR "Autism Spectrum Disorder"[Mesh] OR "Intelligence"[Mesh] OR "Social Support"[Mesh] OR "Adaptation, Psychological"[Mesh] OR "Personality"[Mesh] OR "Quality of Life"[Mesh] OR anxiety[tiab] OR stress*[tiab] OR mental disorder*[tiab] OR PTSD[tiab] OR mental health[tiab] OR depression*[tiab] OR autism*[tiab] OR intelligence[tiab] OR shame[tiab] OR sadness[tiab] OR distress*[tiab] OR well-being[tiab] OR wellbeing[tiab] OR financial[tiab] OR psychosocial[tiab] OR psychological[tiab] OR social support*[tiab] OR professional support*[tiab] OR information need*[tiab] OR support need*[tiab] OR "desire to have children"[tiab] OR child wish[tiab] OR survival guilt*[tiab] OR offspring guilt*[tiab] OR "family communication"[tiab] OR coping*[tiab] OR personality[tiab] OR mental adjustment*[tiab] OR quality of life[tiab]) |
| #3 | #1 AND #2 |
| #4 | (("Animals"[MeSH Terms] OR "models, animal"[MeSH Terms] OR "Animal Experimentation"[MeSH Terms] OR "mice"[tiab] OR "mouse"[tiab] OR "murine"[tiab] OR "rat"[tiab] OR "rats"[tiab]) NOT "Humans"[MeSH Terms]) |
| #5 | #3 NOT #4 |

EMBASE (OVID):

Database(s): Embase Classic+Embase 1947 to 2024 May 20

| # | Searches EMBASE (OVID) |
| --- | --- |
| 1 | exp adenomatous polyp/ or APC protein/ |
| 2 | (adenomatous polyposis col* or APC gene* or protein FAP or familial adenomatous polypos* or familial polyposis col* or adenomatous intestinal polypos* or familial intestinal polypos*).ti,ab,kf. |
| 3 | 1 or 2 |
| 4 | exp mental disease/ or exp anxiety/ or exp anxiety disorder/ or autism/ or emotional disorder/ or exp mood disorder/ or psychosomatic disorder/ or psychotrauma/ or distress syndrome/ or psychological well-being/ or mental health/ or wellbeing/ or psychological aspect/ or exp depression/ or sadness/ or exp physiological stress/ or guilt/ or exp shame/ or exp posttraumatic stress disorder/ or exp mental health/ or psychological aspect/ or exp autism/ or exp intelligence/ or exp social support/ or exp coping behavior/ or exp personality/ or exp "quality of life"/ |
| 5 | (anxiety or stress* or mental disorder* or PTSD or mental health or depression* or autism* or intelligence or shame or sadness or distress* or well-being or wellbeing or financial or psychosocial or psychological or social support* or professional support* or information need* or support need* or "desire to have children" or child wish or survival guilt* or offspring guilt* or "family communication" or coping* or personality or mental adjustment* or quality of life).ti,ab,kf. |
| 6 | 4 or 5 |
| 7 | 3 and 6 |
| 8 | (exp animal/ or exp animal experiment/ or exp animal model/ or exp veterinary medicine/ or (animal* or rodent* or rat or rats or mice or mouse or murine).ti,ab,kw.) not human/ |
| 9 | 7 not 8 |

Cochrane Library (Wiley)

| # | Searches Cochrane Database of Systematic Reviews ; Cochrane Central Register of Controlled Trials |
| --- | --- |
| #1 | MeSH descriptor: [Adenomatous Polyposis Coli] explode all trees |
| #2 | MeSH descriptor: [Genes, APC] explode all trees |
| #3 | MeSH descriptor: [Adenomatous Polyposis Coli Protein] explode all trees |
| #4 | (adenomatous polyposis col* or APC gene* or protein FAP or familial adenomatous polypos* or familial polyposis col* or adenomatous intestinal polypos* or familial intestinal polypos*):ti,ab,kw |
| #5 | #1 or #2 or #3 or #4 |
| #6 | MeSH descriptor: [Mental Disorders] explode all trees |
| #7 | (anxiety or stress* or mental disorder* or PTSD or mental health or depression* or autism* or intelligence or shame or sadness or distress* or well-being or wellbeing or "desire to have children" or child wish or survival guilt* or offspring guilt* or "family communication help" or coping* or personality or mental adjustment* or quality of life or psychosocial impact or mental health symptom* or psychosocial distress or psychological symptom* or psychological burden or financial situation or psychological complain* or psychological support or social support or professional support or information need* or support need*):ti,ab,kw |
| #8 | #6 or #7 |
| #9 | #5 and #8 |

Appendix B: Characteristics of included studies

Table 1.

| *Author and year* | *Country* | *Type of study* | *N* | *Participants* | *Outcomes in study* |
| --- | --- | --- | --- | --- | --- |
| Andrews et al. 2007 | Australia | Cs | 88 | Young adults aged 18 to 35 years | Adjustment problems: Distress |
| Azofra et al. 2016 | USA | CC | 6 | 3 FAP vs 3 healthy controls | Cognitive differences |
| Barber et al. 1986. | Germany | Cr | 1 | Intellectual disability with FAP and childhood autism (pervasive developmental disorder) | Intellectual disability |
| Cali et al. 2021 | USA | CC | 34 | 18 genetically confirmed* FAP vs 16 healthy control | Cognitive differences |
| Codori et al. 1996 | USA | CC | 41 | Children and their parents | Adjustment problems: Anxiety, depression |
| Codori et al. 2003 | USA | CC | 48 | Children | Adjustment problems: Depression, anxiety |
| Cross et al. 1992 | UK | Cr | 1 | Mild intellectual disability with FAP | Intellectual disability |
| Cruz-Correa et al. 2020 | Puerto Rico | CC | 51 | 26 genetically confirmed FAP vs 25 control healthy | Cognitive differences |
| Cui et al. 2005 | China | CC | 12, 89, 163 | **DNA microarray analysis:** 12, 6 schizophrenic patients, 6 healthy controls. **Real-time quantitative RT-PCR:** 89, 59 schizophrenia and schizophrenia-like disorder patients (29 first-episode schizophrenic patients never used antipsychotic medication and 30 relapse patients who used antipsychotic medication), and 30 healthy controls **Linkage disequilibrium analysis:** 163 parent–offspring trios (fathers, mothers, and affected offspring with schizophrenia) | co-occurrent psychiatric disorders: schizophrenia |
| Douma et al. 2010 | The Netherlands | Cs | 525 | FAP diagnosis, 50% risk or proven non-carrier. Mix of clinical^∇^ and genetic diagnosis | Adjustment problems: Distress |
| Douma et al. 2011 | The Netherlands | Cs | 129 | Partners of individuals with FAP. Mix of clinical and genetic diagnosis | Adjustment problems: Distress |
| Dudok DeWit et al. 1998 | The Netherlands | Cs | 45 | Dutch participants at risk for FAP and 18 partners of participants at risk for FAP | Adjustment problems: Anxiety and depression |
| Dudok DeWit et al. 1997 | The Netherlands | Cs | 63 | 45 Dutch participants at risk for FAP and 18 of their partners | Adjustment problems: Distress |
| Dudok DeWit et al. 1998 | The Netherlands | Cs | 23 | people being tested for FAP. (7 pos, 16 neg | Adjustment problems: Distress, anxiety, depression |
| Durno et al. 2012 | Canada | Cs | 32 | FAP with early age colectomy | Adjustment problems: Distress |
| Finch et al. 2005 | USA | Cr | 1 | Intellectual disability with FAP | Intellectual disability |
| Gjone et al. 2011 | Norway | In | 22 | Children / adolescents at risk of FAP. Mix of clinical and genetic diagnosis | Adjustment problems: Distress, co-occurrent psychiatric disorders: general |
| Gorrepati et al. 2018 | USA | Co | 33 | FAP-patients with having undergone an IPAA | Co-occurrent psychiatric disorders: GAD and MDD |
| Heald et al. 2007 | USA | Cr | 1 | Intellectual disability with FAP | Intellectual disability |
| Herrera et al. 1986 | USA | Cr | 1 | Intellectual disability with Gardner syndrome | Intellectual disability |
| Hockey et al. 1989 | Australia | Cr | 2 | Two brothers with intellectual disability. Mother had ID as well. | Intellectual disability |
| Hodgson et al. 1992 | UK | Cr | 2 | Mild intellectual disability in both probands | Intellectual disability |
| Karstensen et al. 2023 | Denmark | Co | 445 vs 1,538 | 445 FAP-patients, 1.538 non-exposed and 1.904 UC patients. Mix of clinical and genetic diagnosis of FAP | Co-occurrent psychiatric disorders and education level |
| Kobayashi et al. 1991 | Japan | Cr | 1 | Intellectual disability with Gardner syndrome | Intellectual disability |
| Levitt et al. 1992 | Canada | Cs | 38 | 38 FAP-patients | Adjustment problems: Depression, Co-occurrent psychiatric disorders: prevalences, Cognitive differences |
| Lindgren et al. 1992 | USA | Cr | 3 | Mild intellectual disability in 13 year old boy, intellectual disability in 8 year old boy and 30 year old woman | Intellectual disability |
| Michie et al. 2001 (first study) | UK | Cs | 208 | 148 adults, 60 children. Both at risk for FAP, having undergone genetic testing since 1990. | Adjustment problems: Distress, anxiety, depression |
| Michie et al. 2001 (second study) | UK | Cs | 31 | Children being tested FAP positive | Adjustment problems: Distress, anxiety, depression |
| Miller et al. 1986 | USA | Cs | 89 | Colectomy + IRA at least 1 year before the trial. | Adjustment problems: Distress, anxiety, Social support |
| Osterfeld et al. 2008 | Germany | Cs | 21 | Clinically proven FAP-patients having undergone prophylactic proctocolectomy | Adjustment problems: Anxiety, depression |
| Pilarski et al. 1999 | USA | Cr | 1 | Intellectual disability in AFAP | Intellectual disability |
| Raedle et al. 2001 | Germany | Cr | 1 | Intellectual disability with FAP | Intellectual disability |
| Wood et al. 2019 | USA | Cs | 79 | Adult FAP-patients. Mix of clinical and genetic diagnosis | Co-occurrent psychiatric disorders: PTSD |
| Yang et al. 2012 | China | CC | 397 vs 473 | 397 MDD vs 473 health controls | Co-occurrent psychiatric disorders: MDD |
| Zhou et al. 2007 | Sweden | CC | 75 vs 476 | 75 ASD vs 476 healthy controls | Co-occurrent psychiatric disorders: ASD |

Cs = cross-sectional, Co = Cohort study, CC = Case Control study, Cr = Case report, FG = Focus groups, In = Interview study, * = FAP diagnosed through genetic testing, ^∇^ = FAP diagnosed based on clinical criteria such as the growth of 100+ colorectal polyps and family history without genetic testing

Appendix C: Result tables

Table 1. Adjustment problems: Distress

| *Author, year and country* | *When in patient journey* | *Measure* | *N* |  | *Outcome* | *Secondary outcome* | *Individual disease characteristics:*  *Polyp burden, surgery, cancer (history) and bowel function* |
| --- | --- | --- | --- | --- | --- | --- | --- |
| Miller et al. 1986, USA | Bowel surgery | Own quest. | 89, 18+, colectomy + IRA at least 1 year before the trial. |  | Almost 40 percent admitted having a high level of concern about future health | 77 percent agreed that “you couldn’t help but feel guilty about passing polyposis on to children.” | All participants had undergone an IRA. Other characteristics not mentioned. |
| Gjone et al. 2011, Norway | Genetic test | CGAS | 22, children / adolescents at risk of FAP |  | A global measure of psychosocial functioning, CGAS, was significantly lower for the parental FAP group (FAP-patients with at least one affected parent) sample: mean 63.6 (SD 13.9) as compared to the GP sample: mean 82.7 (SD 11.3), p 0.000. This indicates a clinically disturbed function (score is below 71). |  | None mentioned. |
| Douma et al. 2010, The Netherlands | Any moment | IES - intrusion  CWS Event: me or my family having FAP† | 525, people with a FAP diagnosis, at 50% risk or a proven non-carrier |  | Mean level of 8.7 on I of IES. Comparable levels to general population. Approximately 20% of the respondents had moderate to severe levels of FAP specific distress that are clinically relevant. 5% scored above 19. Significantly more individuals with a FAP diagnosis had frequent cancer worries than those at risk of FAP or non-carriers. Small subset of increased distress cases. Both history of surgery and personal cancer history were not associated significantly with cancer worries in multivariate analyses. |  | Time since last surgery:  No surgery, because non-carrier 134 (N) (26%)  No surgery (yet) 95 (18%)  0–1 year 16 (3%)  1–2 years 16 (3%)  2–5 years 33 (6%)  5–10 years 73 (14%)  > 10 years 158 (30%)  Personal cancer history: 45 (9%)  Polyp burden and bowel function not mentioned. |
| Douma et al. 2011, The Netherlands | Any moment | IES - intrusion CWS Event: me or my family having FAP† | 129, partners of individuals with FAP |  | Mean FAP-specific distress (IES: 5.6 Intrusion). CWS: 13.6. 30% of the partners reported clinically relevant levels of distress. The FAP-patient having had cancer  (b=0.245), having children (b=0.163), having  higher levels of cancer-specific distress (b=0.288),  and the partner having more feelings of guilt  (b=0.290) toward the patient were associated  significantly with the partners’ cancer-specific  distress. | Stress levels and cancer worries are significantly correlated between FAP-patients and partners. Clinically relevant levels of FAP-specific distress were observed in 56% of the patient-partner dyads. | Personal cancer history:  partners: 4/129  FAP-patients: 43/129 Other characteristics not mentioned. |
| Durno et al. 2012, Canada | After early bowel surgery | IES Event: surgery for FAP† | 32, FAP-patients who underwent a colectomy at an early age |  | +-1/3 (N = 10) was worried about risk of developing cancer postcolectomy. 12% (N = 3) reported “often” or “a lot” of worries and this significantly affected their mood. Again, majority doesn’t experience significant distress, small subgroup does. There is a subgroup of patients (n 3), currently less than 18 years of age, who experience significant levels of distress, as indicated by levels of IES total score (score >25) as well as the intrusion (score>10) and avoidance subscale (score >10). |  | Polyp burden not mentioned. Having a stoma or desmoid tumors were exclusion criteria. No difference in stool frequency between IRA and IPAA. IRA was less continent during the night and day. |
| Michie et al. 2001, UK (second study) | Genetic test | IES Event: poly-posis in the family† | 31, children having tested FAP positive |  | Children did not show increased emotional distress after receiving positive test results. Children showed a decrease in situational distress after a negative test result. At the second post-test assessment, those receiving a positive result were more distressed about FAP in the family than were those receiving a negative test result |  | None mentioned. |
| Andrews et al. 2007, Australia | Genetic test | IES Event: diag-nosed / at risk of FAP† | 88, young adults aged 18 to 35 years |  | Mean scores of 11.4 (IES: 4.7 Intrusion and 6.7 Avoidance). 4.5 and 11.4 percent of participants had scores on the intrusion and avoidance subscales of the IES, respectively, indicative of a significant stress response (Scores > 20). |  | 57/71 had IRA or IPAA. 33 IRA, 21 IPAA, 1 pouch, 2 don't know. Cancer history is measured, also in family, not reported. Polyp burden and bowel function not mentioned. |
| DudokdeWit et al. 1997, The Netherlands | Genetic test | IES Event: disease in the family† | 63, 45 at risk for FAP, 18 partners of at risk for FAP |  | The participants at risk of FAP are under the cut-off value of 16 (4.0 + 4.4 = 8.4) for distress according to the IES indicating non-significant levels of distress. The partners scored under the cut-off value of 8 (3.3 + 3.3 = 6.6) also indicating a subclinical range of distress. |  | None mentioned. |
| DudokdeWit et al. 1998, The Netherlands | Genetic test | IES Event: disease in the family† | 23, people being tested for FAP. (7 pos, 16 neg) |  | Distress (Intrusion and Avoidance) levels at the second time-point in people at risk of FAP were a mean of 5.7, intrusion: 3.0 (SD = 3.8), avoidance: 2.7 (SD = 4.1)) were under the cut-off score of 8, indicating subclinical levels of distress. |  | None mentioned. |
| Michie et al. 2001, UK (first study) | Genetic test | IES Event polyposis in the family† | 208, (148 adults, 60 children) at risk for FAP having undergone genetic testing since 1990 |  | No differences were found on situational distress in both children and adults |  | None mentioned. |

† The impact of event scale is required to be linked to a specific traumatic event. For clinical relevant stress scores of the IES we used a cut-off value of 9 per subscale as was used by Douma et al^(6)^.

Green = No significant result

Yellow = Conflicting results

Blue = Only significant on subgroup level

Red = Significant resultTable 2. Adjustment problems: Anxiety

| *Author, year and country* | *When in patient journey* | *Measure* | *N* |  | *Outcome* | *Secondary outcome* | *Individual disease characteristics:*  *Polyp burden, surgery, cancer (history) and bowel function* |
| --- | --- | --- | --- | --- | --- | --- | --- |
| Michie et al. 2001, UK (first study) | Genetic test | STAI | 208, (148 adults, 60 children) at risk for FAP having undergone genetic testing since 1990 |  | Positive test result: anxiety for children and adults. Worse for adults. Children in general: anxiety was in the normal range. Those receiving positive results experienced anxiety within the clinical ranges (19%), though not significantly different from those receiving negative results, (3%). Adults: receiving positive results were highly anxious, with mean scores and 43% of the group in the clinical range. Mean scores were higher both than the norm and the scores of those receiving negative results. The only difference between children and adults was that, among those with positive results, children were less anxious than adults and fewer children than adults had anxiety scores in the clinical range (19% vs. 43%) | The highest proportion of cases of clinical anxiety when test results are positive and self-esteem and optimism are low (60% and 56% respectively).  (The children’s version of) the Spielberger State Trait Anxiety Inventory | None mentioned. |
| Miller et al. 1986, USA | Bowel surgery | Own quest. | 89, colectomy + IRA at least 1 year before trial |  | Initial responses to the diagnosis,: anxiety (52%), fear of death (31%), |  | All participants had undergone an IRA. Other characteristics not mentioned. |
| Osterfeld et al. 2008, Germany | Bowel surgery | HADS | 21, FAP-patients having undergone prophylactic proctocolectomy |  | Anxiety as measured by the HADS remained unchanged over time; at no time period did patients’ mean levels differ from those of the normal population after adjustment for sex and age. | 38 percent reported considerable fear and depressed mood shortly before the operation, and nearly all patients (90 percent) were afraid of the ileostomy. | Participants had undergone prophylactic proctocolectomy, had no signs of bowel cancer. 3 months after ileostomy closure, 52% reported having adapted to pouch <6 weeks, 29% 8-12 weeks. High stool frequencies were disturbing for most patients. Polyp burden not mentioned. |
| Codori et al, 1996, USA | Genetic test | RCMAS | 41, children and their parents |  | All subclinical. Children Anxiety Scores: regardless of test result, children with affected mothers had increased scores at follow-up; children with affected fathers had decreased scores at follow-up. |  | Polyp burden not mentioned. Other characteristics were not applicable. |
| Codori et al, 2003, USA | Genetic test | RCMAS | 48, children |  | As a group, neither the children nor the parents showed clinically significant psychiatric levels of anxiety at any evaluation time point during the 2–4-year follow-up period. | Thus, although most individual children had no clinically significant declines in their psychological functioning after genetic testing for FAP, the mutation-negative siblings of children who tested positive appear particularly vulnerable to clinical levels of anxiety symptoms after testing. It is noteworthy that testing positive and having a positive sibling is associated with significant, but subclinical, increases in depression symptoms; testing negative and having a positive sibling is associated with clinical elevations in anxiety symptoms. | None mentioned. |
| Michie et al. 2001, UK (second study) | Genetic test | SSTAI | 31, children being having tested positively for FAP positive |  | Anxiety scores were within the normal range and did not change in anxiety levels before and after testing . | Children showed a decrease in anxiety after receiving a negative test result. At the second post-test assessment, those receiving a positive result were more anxious than were those receiving a negative result. | None mentioned. |
| DudokdeWit et al. 1998, The Netherlands | Genetic test | HADS | 45 Dutch participants at risk for FAP and 18 partners of participants at risk for FAP |  | Scores for people at risk of FAP were: anxiety: 5.3 (0.9). Subclinical scores and according to the HADS cut-off scores indicate non-cases of anxiety. |  | None mentioned. |
| DudokdeWit et al. 1998, The Netherlands | Genetic test | HADS | 23, people being tested for FAP. (7 pos, 16 neg) |  | Anxiety levels in people at risk of FAP were a mean of 5.7 (SD = 4.4 ) indicating non-cases of anxiety. |  | None mentioned. |

Green = No significant result

Yellow = Conflicting results

Blue = Only significant on subgroup level

Red = Significant result

Table 3. Adjustment problems: Depression

| *Author, year and country* | *When in patient journey* | *Measure* | *N* |  | *Outcome* | *Secondary outcome* | *Individual disease characteristics:*  *Polyp burden, surgery, cancer (history) and bowel function* |
| --- | --- | --- | --- | --- | --- | --- | --- |
| Codori et al. 2003, USA | Genetic Test | CDI, RADS and BDI | 48, children |  | As a group, neither the children nor the parents showed psychiatric depression levels at any evaluation time point during the 2–4-year follow-up period.  Small minority of groups having elevated depression scores. Testing positive and having a positive sibling is associated with significant, but subclinical, increases in depression symptoms. | Having a sibling who tested positive was associated with statistically significant changes in depression symptoms  It is noteworthy that testing positive and having a positive sibling is associated with significant, but subclinical, increases in depression symptoms | None mentioned. |
| Osterfeld et al. 2008, Germany | Bowel surgery | HADS | 21, FAP-patients having undergone prophylactic proctocolectomy |  | Anxiety and depression as measured by the HADS remained unchanged over time; at no time period did patients’ mean levels differ from those of the normal population after adjustment for sex and age. | 38 percent reported considerable fear and depressed mood shortly before the operation, and nearly all patients (90 percent) were afraid of the ileostomy. | Participants had undergone prophylactic proctocolectomy (IPAA), had no signs of bowel cancer. 3 months after ileostomy closure, 52% reported having adapted to pouch <6 weeks, 29% 8-12 weeks. High stool frequencies were disturbing for most patients. Polyp burden not mentioned. |
| Codori et al. 1996, USA | Genetic Test | CDI, RADS and BDI | 41, children and their parents |  | All subclinical. | Kids: Mutation-positive children with affected mothers had increased depression scores at follow-up: mutation-positive children with affected fathers had decreased depression scores at follow-up.  Parents depression scores:  Unaffected parents in the combined positive and negative children group had increased depression scores at follow-up.  Affected parents had no significant differences in depression scores for only positive, only negative or combined positive and negative children. Unaffected parents also had no significant change in the only positive or only negative children groups. | Polyp burden not mentioned. Other characteristics were not applicable. |
| Dudok- de Wit et al. 1998, The Netherlands | Genetic Test | HADS | 45 Dutch participants at risk for FAP and 18 partners of participants at risk for FAP |  | Scores for people at risk of FAP were depression: 2.4 (0.3). Subclinical score and according to the HADS cut-off scores indicate non-cases of depression. |  | None mentioned. |
| Dudok- de Wit et al. 1998, The Netherlands | Genetic Test | HADS | 23, people being tested for FAP. (7 pos, 16 neg) |  | Depression levels in people at risk of FAP were a mean of 2.2 (SD = 2.2) indicate non-cases of depression. |  | None mentioned. |
| Levitt et al. 1992, Canada | Any moment | BDI | 38, FAP-patients |  | FAP-patients scored in the normal range of depression according to the DBI. |  | None mentioned. |
| Michie et al. 2001, UK (first study) | Genetic Test | HADS | 208, (148 adults, 60 children) at risk for FAP having undergone genetic testing since 1990 |  | Depression was in the normal range for both children and adults and did not change over time.  There was no difference between groups in depression at any time point. |  | None mentioned. |

Green = No significant result

Yellow = Conflicting results

Blue = Only significant on subgroup level

Red = Significant result

Table 4. Co-occurrent psychiatric disorders

| *Author, year and country* | *co-occurrent psychiatric disorders* | *Assessed by* | *N* |  | *Outcome* | *Secondary outcome* | *Individual disease characteristics:*  *Polyp burden, surgery, cancer (history) and bowel function* |
| --- | --- | --- | --- | --- | --- | --- | --- |
| Cui et al. 2005, China | Schizophrenia | DNA microarray analyses, Real-time quantitative RT-PCR and Linkage disequilibrium analysis | 12 6vs6), for identifying markers, 89 (59vs30), 163 parent offspring trios |  | Three SNIP’s all showed significant association with schizophrenia.  We found a significant association between the *APC* haplotypes, rs2229992–rs42427–rs465899, and schizophrenia (Global w2 = 44.376, df = 7, P < 0.001). |  | Not applicable. |
| Wood et al. 2019, USA | PTSD | Scored by authors by number of symptoms | 79, adult FAP-patients |  | - Twenty two had no psychosocial symptoms at all, whereas 57 did (72.2%).  - Nine patients (11.4%) fit all 4 of the DSM-5 criteria for PTSD, and 8 (10.1%) fit 3 of them.  - Women are affected with mental health issues more commonly than men both overall and in the group with PTSD-like and PPTSD-like symptoms.  - Patients endorsed an average of 4.3 mental health symptoms each, with a range from 1 to 14. PTSD-like patients had an average of 9.3 psychosocial symptoms each, compared with 8.3 for PPTSD-like and 2.3 for non- PTSD-like designated patients  - Death or serious injury in the family was an almost universal factor in patients with PTSD/PPTSD.  - Symptomatic desmoid disease was present in 10 (59%) of the 17 PTSD/PPTSD patients  - The incidence of PTSD/PPTSD is most strongly related to the type of surgery performed (IPAA vs IRA) and the occurrence of complications. | Psychosocial symptoms are amongst others (patients endorsing symptoms vs patients not endorsing symptoms in %)  - Anxiety, (42% vs 76%)  - fear, (42% vs 53%)  - depression, (24% vs 59%)  - social anxiety, (14% vs 47%)  - extreme stress, (13% vs 29%)  - guilt and (13% vs 47%)  - suicidal thoughts. (8% vs 35%) | The incidence  of PTSD/PPTSD is most strongly related to the type of surgery performed (p<0.05) where IPAA had the most PTSD/PPTSD incidence. Other characteristics not mentioned. |
| Yang et al. 2012, China | MDD | Genotype and allellfrequency | 397 MDD vs 473 |  | Two SNPs (rs2464805 and rs563556) within the *APC* gene exhibited a statistically significant association with MDD when analyzed by genotype and allele frequencies.  The minor allele frequency for rs2464805 was higher for cases compared to controls and for rs563556 this the minor allele frequency was lower. |  | Not applicable. |
| Zhou et al. 2007, Sweden | ASD | Genotype and allel frequency | 75 ASD vs 476 healthy controls |  | One SNP, 8636C>A (rs1804197) in the 3’-untranslated region of the adenomatous polyposis coli (*APC*) gene was associated with autism spectrum disorder (ASD) |  | Not applicable. |
| Gjone et al. 2011, Norway | Co-occurrent psychiatric disorders | As rated by the first and second author of the study using the DSM-IV (Inter-rater reliability was .8; very good). | 22, children / adolescents at risk for FAP |  | In a nationwide sample of children and adolescents aged 11–20 years where one parent had FAP, significantly more adolescents fulfilled criteria for psychiatric diagnoses in the age group above 15 in the parFAP sample than in a general population sample.  parFAP: 8/22 (37%) had psychiatric disorder.  GP: 5/35 (14%) had psychiatric disorder |  | None mentioned |
| Karstensen et al. 2023, Denmark | Co-occurrent psychiatric disorders | Prevalence psychiatric contacts, psychiatric prescriptions and psychiatric diagnoses. | 445, FAP-patients, 1538 non-exposed and 1904 Ulcerative Colitis (UC) patients |  | More psychiatric contacts (HR: 1.76), psychiatric prescriptions (HR: 1.52), psychiatric diagnoses (HR:1.72), psychiatric event (HR:1.55) for FAP compared to non-exposed individuals. For FAP-patients, after adjusting for sex and educational level, we failed to find a significant association between colectomy and the risk of any psychiatric event. When probands and callups were compared separately to non-exposed individuals, probands did not have a significantly increased risk of psychiatric events, psychiatric diagnoses, needs for psychiatrics prescriptions, or psychiatric contacts, while these risks were significantly increased for callups (Table 4). | When adjusting for age, sex and the event of cancer, the risk of a diagnosis for a mood or behavioural and emotional disorder was significantly higher for FAP-patients than nonexposed individuals. For FAP-patients, the median age for the diagnosis of a mood disorder or a behavioural and emotional disorder was 32.7 years (range 15.0-73.6 years) and 14.7 (range 5.5-47.7 years), respectively. No significant difference was found for schizophrenia, neurotic, or developmental disorders. While the risk of mood or neurotic disorders were significantly increased for call-ups, this was not the case for probands. | Authors mention polyp burden is not taken into account. Other characteristics not mentioned. |
| Levitt et al. 1992, Canada | Co-occurrent psychiatric disorders | Interview, not official records. | 38 vs 19 |  | 13 of 38 had any disorder (34%). Major Depression: N = 2, 7%. Minor depression: N = 6, 16%. Bipolar disorder: N = 0, 0%. Generalized anxiety disorder: N = 4, 10%, Panic disorder: N = 0, 0%, Schizophrenia: N = 2, 5% and any disorder: N = 13, 34% |  | None mentioned. |
| Gorrepati et al. 2018, USA | GAD or MDD | Formal diagnoses | 33 |  | 4 of the 33 FAP-patients had anxiety and/or depression. (12,1%). Not significantly different from the general population |  | FAP patients with having undergone IPAA. Other characteristics not mentioned. |

ASD = Autism Spectrum Disorder; MDD = Major Depressive Disorder; GAD = Generalized Anxiety Disorder; PTSD = Post-traumatic stress disorder

Green = No significant result

Yellow = Conflicting results

Red = Significant result

Table 5. Cognitive differences

| *Author, year and country* | *Topic* | *Population* | *N* |  | *Outcome* | *Secondary outcome* | *Individual disease characteristics:*  *Polyp burden, surgery, cancer (history) and bowel function* |
| --- | --- | --- | --- | --- | --- | --- | --- |
| Azofra et al. 2016, America | White matter and IQ | Age 20-27 | 6 (3 FAP, 3 control) |  | • No differences were found in the white matter matrices in all three pairs between the FAP-patients and the non-FAP siblings.  • The sibling pairs scored similarly on the WASI-II (general intelligence), D-KEFS (executive function), and WIAT-III (academic skills) tests, with only a single notable difference in family 2, where the patient with FAP scored five points lower than his sibling pair on the design fluency portion of the D-KEFS test, corresponding to a difference of 1.67 standard deviations. |  | Pers 1: First colonoscopy  at age ten, multiple sessile adenomas, no surgery. No cancer. Pers 2: Polyp burden not mentioned. IPAA, No cancer. Pers 3: endoscopy at 14: more than 100 gastric polyps and more than 200 colonic polyps, IPAA, No cancer. Bowel function not mentioned throughout. |
| Levitt et al. 1992, Canada | IQ: DS + subtest of the WAIS | Mean age 39.5, sd 11. | 38 |  | Scores on the Shipley Institute of Living Scale (SILS) and the Digit Symbols (DS) subtest of the WAIS are on average level. | Isolated FAP seems to score somewhat higher than the inherited FAP group. | None mentioned. |
| Cali et al. 2021, USA | Resting state networks and general cognitive score (Batería III Woodcock M~unez) | Age 10+ | 34 (18 FAP, 16 control) |  | All eight resting state networks were noted as the most identifiable networks  where significant differences were present.  When adjusting for cognition as a covariate to the rsfMRI data, significant differences were virtually eliminated, with only indications of minor differences throughout the components (Fig. 2, right). This suggests a high correlation between cognitive functioning and rsfMRI findings. | Additionally, a secondary analysis of the FAP group revealed no significant differences when accounting for genotype-phenotype severity using “attenuated” and “classic” FAP as covariate groupings. | None mentioned. |
| Cruz-Correa et al. 2020, Puerto Rico | IQ | Age 10+, and no previous diagnosis of any major psychiatric condition given | 51 (26 FAP, 25 control) |  | FAP cases and controls fell within the average range of functioning, although the FAP-patients had statistically significantly lower functioning compared with age-, gender- and education-matched controls.  Batería-III: across all summary scores, FAP cases had significantly more risk for cognitive dysfunction than the controls, with the cases showing performances in the lower quartile on Verbal Ability, Thinking Ability, Cognitive Efficiency, Long-Term Retrieval, Processing Speed, Phonemic Awareness, Working Memory, Cognitive Fluency, and Executive Processes. FAP cases scored within the deficient range for Long-Term Retrieval and Cognitive Fluency.  Specific group comparisons on the obtained scores revealed a similar pattern of findings wherein the FAP Group performed significantly lower than controls on: Verbal Ability; Thinking Ability; Cognitive Efficiency; Long-Term Memory; Processing Speed; Phonemic Awareness; Working Memory; Cognitive Fluency; and Executive Processes. After Bonferonni Correction, nine of the Batería-III subtests remained statistically significant including: Verbal Comprehension, Visual-Auditory Learning, Spatial Relations, Sound Blending, Concept Formation, Visual Matching, Auditory Working Memory, Retrieval Fluency, and Decision Speed. | AFAP vs FAP:  Findings revealed that the two FAP groups were not significantly different on IQ (p = 0.709). However, FAP cases with mutations located within the classic mutation cluster had marginally lower performance on Woodcock-Muñoz summary scores of Cognitive Efficiency (p = 0.076) and Processing Speed, (p = 0.056), and on BRIEF Adult scales of Shift (p = 0.058) and Initiate (p = 0.081) compared to FAP cases with mutations in the attenuated FAP region (Table 3). | 3 out of 26 cases had had chemotherapy for colon cancer. Other characteristics not mentioned. |
| Karstensen et al. 2023, Denmark | Education level | Education level | 445, FAP-patients, 1538 non-exposed and 1904 Ulcerative Colitis (UC) patients |  | The highest attained level of education was significantly lower for FAP-patients than nonexposed. In call-ups, 44.9% and 21.1% had a primary/lower secondary and bachelor/master/doctoral education, respectively, while the rates for probands were 35.2% and 32.4%, respectively (Figure 2). However, this was not significant (p=0.069). Excluding FAP-patients without a known genotype did not change these results.  While the educational level interacted significantly with the risk on any psychiatric event and risk of psychiatric diagnosis, it did not interact with need for a psychiatric prescription or contacts (Supplementary Material 4). Additionally, we found that the educational level was significantly different (p=0.008) for FAP-patients with a colectomy than those without a colectomy. |  | Authors mention polyp burden is not taken into account. Other characteristics not mentioned. |

Green = No significant result

Yellow = Conflicting results

Red = Significant result

Appendix D: Quality assessment

Table 1. Axis cross-sectional. The AXIS focusses on the aims of the study, the appropriateness of the study design, sample size and representation of the sample, selection bias, non-responders, measurement validity and reliability, statistics, limitations and ethical approval/consent of participants.

| Study | Intro. | Methods | Results | Discussion | Total | Extra comments |
| --- | --- | --- | --- | --- | --- | --- |
| Andrews et al. 2007 | 1/1 | 7/11 | 3/5 | 2/2 | 14/20 | Potential selection bias, no validation of questionnaire, no non-responders described. |
| Douma et al. 2010 | 1/1 | 11/11 | 5/5 | 2/2 | 20/20 | No reason for suspicion of bias |
| Douma et al. 2011 | 1/1 | 11/11 | 5/5 | 2/2 | 20/20 | Excellent, differences were found in participants vs. non-participants. Conclusions need to be drawn with caution |
| DudokdeWit et al. 1998 | 1/1 | 9/11 | 4/5 | 2/2 | 17/20 | Sample size was not large, non-responders weren't categorized, caution with generalization |
| DudokdeWit et al. 1997 | 1/1 | 9/11 | 4/5 | 2/2 | 17/20 | Small sample size, non-responders weren't categorized, caution with generalization |
| DudokdeWit et al. 1998 | 1/1 | 10/11 | 5/5 | 2/2 | 19/20 | Sample size was not large, people that didn't complete the follow up were on average higher educated than participants completing the follow up; caution with generalization |
| Durno et al. 2012 | 1/1 | 10/11 | 4/5 | 2/2 | 18/20 | Overall fine bias assessment. Non-responders percentage was low, but no reason for non-compliance was given |
| Levitt et al. 1992 | 1/1 | 10/11 | 5/5 | 2/2 | 19/20 | Overall good. Possible selection bias. Isolated FAP label might be speculative, since people in the family might not be recognized as FAP-patients by the study. Psychiatric diagnoses assessed by an interview, not official records. |
| Michie et al. 2001 | 1/1 | 10/11 | 3/5 | 2/2 | 17/20 | Good quality, non-responders not addressed. Careful with generalization |
| Miller et al. 1986 | 1/1 | 10/11 | 4/5 | 2/2 | 18/20 | Good quality, non-responders not addressed. Careful with generalization |
| Osterfeld et al. 2008 | 1/1 | 9/11 | 4/5 | 2/2 | 17/20 | Mediocre, possible skewed results: no prior mental condition of patients included. Non-responders not described. However response rate is high |
| Wood et al. 2019 | 1/1 | 9/11 | 3/5 | 2/2 | 16/20 | Mediocre. Questionnaire wasn't validated. Response rate was low, possible higher response of people endorsing psychosocial symptoms, non-responders not described. PTSD wasn't formally diagnosed. Authors scored symptoms themselves. |

Table 2. NOS for case control studies. The NOS rates the risk of bias in case-controls studies on three domains: selection, comparability and exposure.

| Study | Selection | Comparability | Exposure | Total | Extra comments |
| --- | --- | --- | --- | --- | --- |
| Azofra et al. 2016 | 3/4 | 2/2 | 3/3 | 8/9 | Generally good, might be prone to selection bias. Since N = 3: weak prove. Partial bias possible at Ascertainment of exposure, scans are secure, parents might be biased. |
| Cali et al. 2021 | 4/4 | 2/2 | 2/3 | 8/9 | Not described where control group was recruited. Response rates also not described. No big influence on the validity. |
| Codori et al. 1996 | 4/4 | 1/2 | 2/3 | 7/9 | Might be influenced by a significant difference in age. Non responses not described. No description of |
| Codori et al. 2003 | 3/4 | 2/2 | 3/3 | 8/9 | Generally good, all from registry, people not at registry could have different levels of psychological problems |
| Cruz-Correa et al. 2020 | 4/4 | 2/2 | 2/3 | 8/9 | Generally good, non-responses not described |
| Cui et al. 2005 | 3/4 | 2/2 | 2/3 | 7/9 | Generally good, non-response rate and selection process not described |
| Gjone et al. 2010 | 4/4 | 2/2 | 1/3 | 7/9 | Mediocre, non-responders rate not described. Interviewers were not blinded. This might lead to some bias |
| Karstensen et al. 2023 | 4/4 | 2/2 | 3/3 | 9/9 | Good quality. Low risk of bias. |
| Yang et al. 2012 | 4/4 | 2/2 | 2/3 | 8/9 | Generally good, non-response rate and selection process not described. Caution with generalization |
| Zhou et al. 2007 | 3/4 | 0/2 | 3/3 | 6/9 | Mediocre, non-response rate and selection process not described. No control variables described. Cases had a high prevalence of comorbidity. Careful with conclusions |

Table 3. NOS for cohort studies. The NOS for cohort studies uses three domains: selection, comparability and outcome.

| Study | Selection | Comparability | Exposure | Total | Extra comments |
| --- | --- | --- | --- | --- | --- |
| Gorrepati et al. 2018 | 4/4 | 2/2 | 3/3 | 9/9 | Good, careful with generalizing because of small N |

Table 4. Checklist Boejie Qualitative. The checklist from Boejie et al(14) focusses on formulation of the research question, data collection techniques, benefits and risks and transferability.

| Study | Haeld et al. 2007 | Raedle et al. 2001 | Finch et al. 2005 | Herrera et al. 1986 | Cross et al. 1992 | Hockey et al. 1989 | Kobayashi et al. 1991 | Lindgren et al. 1992 | Pilarski et al. 1999 | Hodgson et al. 1992 | Barber et al. 1986 |
| --- | --- | --- | --- | --- | --- | --- | --- | --- | --- | --- | --- |
| 1. Research purposes or questions are linked to the problem and/or to the review of the literature. | 2 | 2 | 2 | 2 | 2 | 1 | 2 | 2 | 2 | 2 | 2 |
| 2. Sample size and configuration fit the purpose and sampling strategy. | 2 | 2 | 2 | 2 | 2 | 2 | 2 | 2 | 2 | 2 | 2 |
| 3. Data collection techniques are correctly used. | 2 | 2 | 2 | 2 | 2 | 2 | 2 | 2 | 2 | 2 | 2 |
| 4. Data analysis techniques are tailored to the reported study. | 2 | 2 | 2 | 2 | 2 | 2 | 2 | 2 | 2 | 2 | 2 |
| 5. Interpretations of data are demonstrably plausible and/or sufficiently substantiated with data. | 2 | 2 | 2 | 2 | 2 | 2 | 2 | 2 | 2 | 2 | 2 |
| 6. The clinical, policy, theoretical, disciplinary, and/or other significance of the findings is thoughtfully considered. | 2 | 2 | 2 | 2 | 2 | 1 | 2 | 2 | 2 | 2 | 2 |
| 7. The study discusses techniques specifically intended to ensure that the study is scientifically and/or ethnographically valid or ‘good’. | 0 | 0 | 2 | 2 | 2 | 2 | 2 | 2 | 2 | 2 | 2 |
| 8. Benefits and risks distinctive to the study are addressed. | 0 | 0 | 0 | 0 | 0 | 0 | 0 | 0 | 0 | 0 | 0 |
| 9. Given the reporting style, elements of the research report are placed where readers are likely to find them. | 2 | 2 | 2 | 2 | 2 | 2 | 2 | 2 | 2 | 2 | 2 |
| 10. The authors provide information regarding participants, setting and context so that the reader might be able to determine the relevance of the findings to other settings (transferability) | 2 | 2 | 2 | 2 | 2 | 2 | 2 | 2 | 2 | 2 | 2 |
| Total score | 16 | 16 | 18 | 18 | 18 | 16 | 18 | 18 | 18 | 18 | 18 |
| *Extra comments* | Quite good, no specific techniques are described | Quite good, no specific techniques are described | Good quality | Good quality | Good quality. Included case report Aunt of proband | Good quality | Good quality | Good quality case report | Good quality. Patient with AFAP. | Good case report | Good quality |

Appendix E: Effect sizes

Table 1.

| Paper | Type of effect size | Effect size | Interpretation |
| --- | --- | --- | --- |
| Cali et al. ^(18)^ IQ | Cohen’s D | -1.66 | Large |
| Cruz-Correa et al. ^(44)^ IQ | Cohen’s D | -0.82 | Large |
| Cruz-Correa et al. ^(44)^ verbal ability | Cohen’s D | -0.97 | Large |
| Cruz-Correa et al. ^(44)^ thinking ability | Cohen’s D | -1.66 | Large |
| Cruz-Correa et al. ^(44)^ cognitive efficiency | Cohen’s D | -1.24 | Large |
| Cruz-Correa et al. ^(44)^ long-term memory | Cohen’s D | -1.38 | Large |
| Cruz-Correa et al. ^(44)^ processing speed | Cohen’s D | -1.35 | Large |
| Cruz-Correa et al. ^(44)^ phonemic awareness | Cohen’s D | -0.92 | Large |
| Cruz-Correa et al. ^(44)^ working memory | Cohen’s D | -1.01 | Large |
| Cruz-Correa et al. ^(44)^ cognitive fluency | Cohen’s D | -1.19 | Large |
| Cruz-Correa et al. ^(44)^ executive processes | Cohen’s D | -1.43 | Large |
| Douma et al. ^(6)^ Cancer Worry Scale FAP-at risk | Cohen’s D | 0.48 | Small |
| Douma et al. ^(6)^ Cancer Worry Scale FAP-non carrier | Cohen’s D | 0.34 | Small |
| Gjone et al. ^(30)^ CGAS | Cohen’s D | -1.55 | Large |
| Michie et al. ^(31)^ (first study) anxiety-adults | Cohen’s D | 0.83 | Large |
| Michie et al. ^(31)^ (first study) anxiety-children | Cohen’s D | 0.52 | Medium |
| Michie et al. ^(31)^ (first study) depression-adults | Cohen’s D | 0.26 | Small |
| Michie et al. ^(31)^ (first study) depression-children | Cohen’s D | 0.54 | Medium |
| Michie et al. ^(31)^ (first study) situational distress-adults | Cohen’s D | 0.34 | Small |
| Michie et al. ^(31)^ (first study) situational distress-Chi | Cohen’s D | .18 | Small |
| Yang et al. ^(19)^ SNP (1 out of 2 | Odds ratio | 1.90 | Medium |
| Yang et al. ^(19)^ SNP (2 out of 2 | Odds ratio | 0.56 | Small |
| Zhou et al. ^(8)^ SNP Genotypes C/A | Odds ratio | 2.20 | Medium |
| Zhou et al. ^(8)^ SNP Alleles A | Odds ratio | 3.40 | Large |
| Karstensen et al. ^(41)^ Psychiatric contact | Odds ratio | 1.76 | Medium |
| Karstensen et al. ^(41)^ Psychiatric prescription | Odds ratio | 1.52 | Medium |
| Karstensen et al. ^(41)^ Receiving psychiatric diagnose | Odds ratio | 1.72 | Medium |
| Karstensen et al. ^(41)^ Risk of psychiatric event | Odds ratio | 1.55 | Medium |

Cohen’s D rule of thumb: Small: < 0.5, Medium: 0.5-0.8, Large: > 0.8; Odds ratio rule of thumb: Small: < 1.5, Medium: 1.5-3, Large: > 3
